# Down-regulation of salt-inducible kinase 1 (SIK1) is mediated by RNF2 in hepatocarcinogenesis

**DOI:** 10.18632/oncotarget.13673

**Published:** 2016-11-29

**Authors:** Chao Qu, Yaqin Qu

**Affiliations:** ^1^ Department of Radiation Oncology, The First Hospital of Jilin University, Changchun, China

**Keywords:** SIK1, RNF2, liver cancer, ubiquitination

## Abstract

Our previous study reported that down-regulation of SIK1 accelerates the growth and invasion of hepatocellular carcinoma (HCC). However, the underlying mechanism leading to SIK1 down-regulation in HCC largely remains to be determined. Herein, we demonstrated that RNF2 expression is negatively correlated with SIK1 levels in HCC tissues. Kaplan-Meier analysis of tumor samples revealed that high RNF2 expression with concurrent low SIK1 expression is associated with poor overall survival. The down-regulation of RNF2 expression in HCC cells significantly reduces tumor cell growth and metastasis, while the simultaneous down-regulation of both RNF2 and SIK1 restores tumor cell growth *in vitro* and in tumor xenograft models. Mechanistically, we identified RNF2 as an E3 ligase that targets SIK1 for degradation. We further demonstrated that direct physical interaction between RNF2 and SIK1 triggers SIK1 down-regulation in HCC cells. These data suggest that RNF2 is an important upstream negative regulator of SIK1 and that restoration of SIK1 levels induced by loss of RNF2 inhibited HCC cell growth and promoted apoptosis, which may represent a promising therapeutic strategy for HCC treatment.

## INTRODUCTION

Hepatocellular carcinoma (HCC) is one of the most death causing cancers among the world [1]. Despite advances in the diagnosis and treatment of HCC, the overall survival of patients with HCC remains poor due to a high rate of recurrence and distant metastasis after hepatectomy [2]. And the understanding of the underlying molecular mechanisms of HCC remains lacking. Thus, it is crucial to explore effective and novel biomarkers for recurrence and metastasis to improve combined treatment strategy for future prognosis. More than 100 putative driver genes had been found to be associated with multiple recurrently altered pathways in HCC and may be potential targets for regulation by therapies [3]. Therefore, it is necessary to elucidate their role and action mechanisms in HCC growth, invasion and metastasis.

Salt-inducible kinase 1 (SIK1) is an AMPK-related serine/threonine kinase, which is activated through the phosphorylation by liver kinase B1 (LKB1) [4]. However, SIK1 is unique among these enzymes by cAMP-dependent transcriptional regulation in several cell types including adrenocortical cells, hepatocytes, and skeletal myoblasts [5]. SIK1 is a founding member of the SIK family of kinases that comprise of SIK1, SIK2, and SIK3 [6]. SIK1 can function as an inhibitory kinase for CRTCs and further hamper subsequent gluconeogenesis in hepatocytes [7]. Besides, it's involved in the regulation of myogenesis through direct phosphorylation of histone deacetylase 5. In addition, the regulation of lipogenesis in the liver indicates the importance of SIK1 regulating metabolic pathways in insulin-sensitive tissues [8, 9]. Recently, other groups and us reported that SIK1 plays a critical role in HCC development [10, 11]. Although SIK1 was rapidly degraded in undifferentiated myoblasts and HCC [12], the mechanisms underlying the rapid, proteasome- dependent degradation of endogenous SIK1 has not been previously reported. Definitely, it is necessary to investigate the molecular mechanisms which contribute to regulated SIK1 degradation.

RNF2, also known as Ring1B/Ring2, is a key component of the polycomb repression complex 1 [13]. RNF2 has been found to regulate different biological processes by distinct molecular mechanisms. For example, RNF2 plays the role of an E3 ligase, and a previous study demonstrated that it acts on histone H2A at lysine 119 [14]. Cdkn2a was found to be another target of RNF2, however, the lethal phenotype of RNF2-deficient mice cannot be rescued by Cdkn2a deletion [15], indicating that other important targets of RNF2 likely exist. It has been reported that RNF2 is highly expressed in many different tumors, and up-regulation of RNF2 may promote tumorigenesis and metastasis [16]. However, the precise mechanism by which RNF2 exerts its effects on human malignant tumors largely remains elusive. Based on these results, we suspected that RNF2 also might potentially play an important role in the regulation of HCC cell proliferation. Further studies are essential to confirm this hypothesis, and precisely illustrate the function and action mechanism of RNF2 in the development and progression of HCC

Given that there is an inverse correlation between RNF2 and SIK1 expression observed in HCC tissues, we hypothesized that RNF2 is very important for SIK1 stability and degradation. In the present study, we found that the expression of RNF2 was significantly increased in human HCC tissues compared with the normal tissues. Upregulation of RNF2 promoted HCC cell proliferation and metastasis. Mechanistic studies reveal that the oncogenic function of RNF2 is at least partially dependent on SIK1 because SIK1 knockdown could reverse this phenotype. Thus, SIK1 is a new target of RNF2 E3 ligase.

## RESULTS

### The inverse expression of SIK1 and RNF2 in human HCC tissues are tightly correlated with clinical outcome

**Figure 1:**
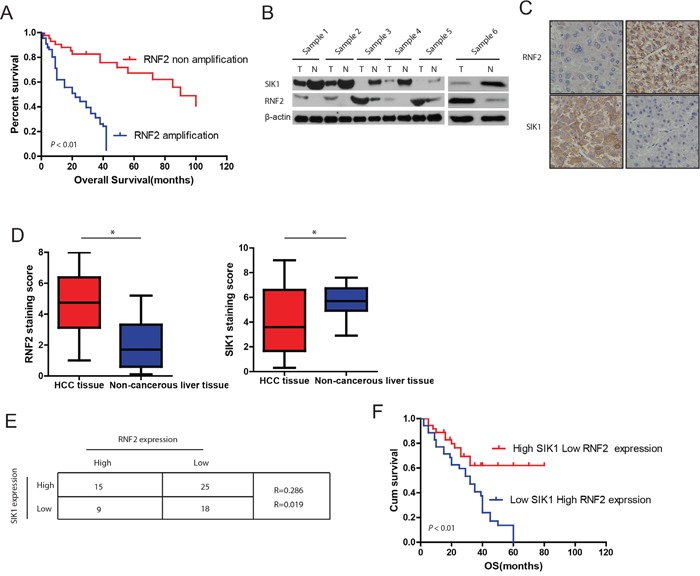
Expression patterns of RNF2 and SIK1 in human HCC **A**. Relationship between RNF2 amplification and HCC prognosis. Kaplan-Meier curves based on data from TCGA show that RNF2 amplification in HCC was associated with shorter overall survival (*P* < 0.05). **B**. RNF2 and SIK1 expression in HCC tissues and paired normal liver tissues. Western blots of RNF2 and SIK1 in six representative paired samples of non-tumor tissue (N) and HCC tissue (T) are shown. β-Actin was used as a control for protein load. **C**. Expression patterns of RNF2 and SIK1 immunoreactivity in HCC samples and in non-tumor tissues from a representative case. **D**. RNF2 expression levels in noncancerous liver tissues were lower than those in HCC tissues (left) (*P* < 0.05). Conversely, SIK1 expression levels in noncancerous liver tissues were higher than those in HCC tissues (right) (*P* < 0.05). **E**. Low SIK1 levels were correlated with high RNF2 expression in HCC (*P* < 0.05). The median expression levels of SIK1 and RNF2 were used as the cutoff values. **F**. Kaplan-Meier curves show that HCC patients with high RNF2 and low SIK1 immunoreactivity had a poorer prognosis than patients with low RNF2 and high SIK1 immunoreactivity did. The data are expressed as the mean ± SD. The results are representative of three independent experiments. *P < 0.05, **P < 0.01.

We previously reported that downregulation of SIK1 markedly promotes HCC cells proliferation, migration, invasion, as well as EMT *in vitro* and increases tumor growth and metastasis in the mouse xenograft model [10]. To further investigate the mechanism by which SIK1 was downregulated in HCC, we sought to identify previously unknown cellular interaction partners of SIK1 by affinity purification and mass-spectrometry (MS) analysis of Flag-tagged-SIK1. The MS analysis indicated that RNF2 was specifically present in complex with Flag-SIK1. Since very little was known about the effect of RNF2 on HCC, we firstly investigated the clinical role of RNF2 in HCC using clinical data from TCGA Data Portal. It was found that the percentages of HCC samples with RNF2 mRNA amplification were 19.7%, implying that the amplification of RNF2 mRNA is associated with poor prognosis in HCC. Kaplan-Meier analysis further confirmed that RNF2 amplification is closely correlated with higher risk in HCC patients (Figure 1A).

To further investigate the expression patterns of RNF2 and SIK1 in HCC, we randomly examined six pairs of human HCC samples and matched non-tumor tissues. It was observed that RNF2 levels in all tested HCC samples, were consistently higher compared with adjacent normal liver tissues. Concurrently, SIK1 levels in tumors were significantly lower compared with levels in adjacent normal liver tissues (Figure 1B). These data indicated that RNF2 overexpression correlates with SIK1 down-regulation in HCC. To further quantitate the expression of RNF2 and SIK1, we used Immunohistochemical staining to analyze another cohort of 67 HCC patients with clinical records by correlating expression levels with overall survival (Supplementary Table 1). Our data further confirmed the inverse correlations between RNF2 and SIK1 in HCC tissues (Figure 1C). The staining score of RNF2 was higher in HCC tissues (4.46 ± 0.13) than in non-tumor tissues (3.02 ± 0.23) (Figure 1D, P < 0.009). Conversely, SIK1 levels were higher in non-tumor tissues (5.15 ± 0.41) than in HCC tissues (3.12 ± 0.29, *P* < 0.006) (Figure 1D). Obviously, RNF2 expression in HCC tissues was inversely correlated with SIK1 levels (Figure 1E; *R* = − 0.304, *P* = 0.013). We further investigated the effect of RNF2 and SIK1 expression pattern on the outcome of HCC patients. The Kaplan-Meier Survival analysis of the 67 patients suggests that expression of high RNF2 level with concurrent low SIK1 tended to correlate with a poor prognosis (Figure 1F).

### Effect of RNF2 on HCC cell proliferation

**Figure 2:**
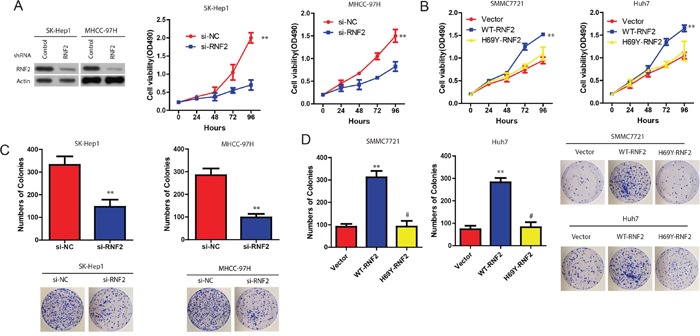
Effect of RNF2 on HCC cell proliferation **A**. Cell viability of SK-Hep1 and MHCC-97H cells when RNF2 was downregulated. **B**. Cell viability of SMCC7721 and Huh cells when RNF2 was overexpressed. **C** and **D**. Colony formation of HCC cells with WT-RNF2 or RNF2-H69Y mutant, or HCC cells with si-RNF2 treatment. The data are expressed as the mean ± SD. The results are representative of three independent experiments. *P < 0.05, **P < 0.01.

The enhancement of RNF2 expression in HCC samples prompted us to explore the possible biological significance of RNF2. We firstly examined the effect of RNF2 on cell proliferation. MTT assay indicated that compared with the controls, the proliferation of SK-Hep1 or MHCC-97H cells was significantly decreased in si-RNF2 transfected cells (Figure 2A). However, the cell growth was significantly enhanced in WT-RNF2, but not RNF2-H69Y (inactivity mutant) SK-Hep1 and MHCC-97H transfected HCC cells (Figure 2B). Consistently, colony-formation assays revealed that RNF2 knockdown significantly decreased clonogenic survival in SK-Hep1 or MHCC-97H cells, while clonogenic survival was enhanced in WT-RNF2, but not in RNF2-H69Y mut-transfected HCC cells (Figure 2C). These data indicate that RNF2 promotes HCC cell proliferation, and this effect is obviously associated with its E3 ligase activity.

### RNF2 promotes HCC cell proliferation by accelerating the cell-cycle progression

**Figure 3:**
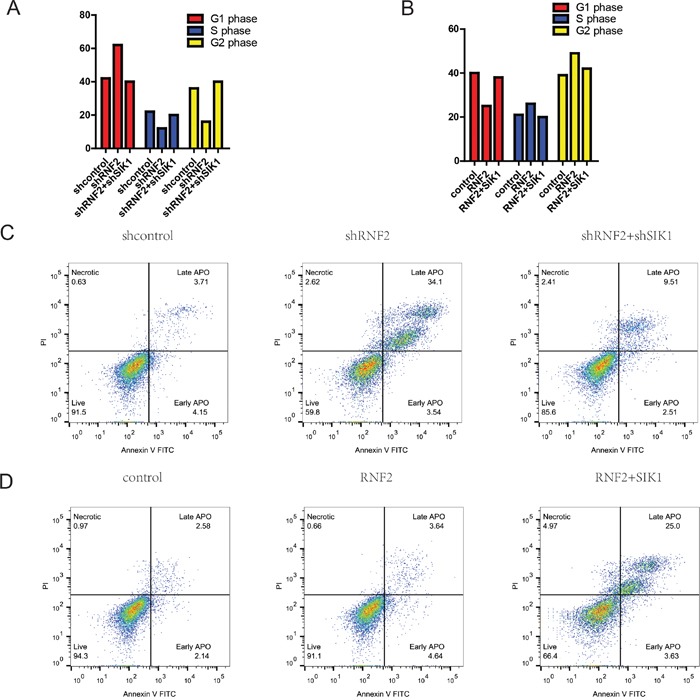
RNF2 promotes HCC cell proliferation by accelerating cell-cycle progression **A** and **B**. Cell cycle analysis of HCC cells treated with sh-RNF2 or HCC cells treated with pCDNA-RNF2-wt for 48 hours. The bar chart represents the percentage of cells in the G1-G0, S, or G2-M phase, as indicated. **C** and **D**. RNF2-knockdown HCC cells conferred a large subgroup of apoptotic cells as compared with control cells, whereas overexpression of RNF2 didn't affect apoptosis as compared with the control. These data are expressed as the mean ± SD. The results are representative of three independent experiments. *P < 0.05, **P < 0.01. Statistical significance was concluded at *P < 0.05, **P < 0.01, ***P < 0.001; # represents no statistical significance.

To further investigate whether the impact of RNF2 on HCC cell proliferation occurs by changing apoptosis or cell cycle progression, flow cytometric assay was performed. Our data indicated that compared with cells that were transfected with scramble, the cell cycle progression of MHCC-97H cells with RNF2 knockdown was significantly stalled at the G0/G1 phase (Figure 3A). Conversely, RNF2 overexpression obviously accelerated the cell cycle progression (Figure 3B).

As determined by flow cytometry assays, after knockdown of RNF2, the percentage of apoptotic HCC cells was significantly elevated (Figure 3C). Upregulation of RNF2 didn't obviously affect the cellular apoptosis ratio. However, SIK1 overexperssion augmented it. (Figure 3D). Collectively, these data clearly demonstrate that RNF2 has a growth-promotive function in HCC.

### RNF2 promotes the invasion and metastasis of HCC cells

**Figure 4:**
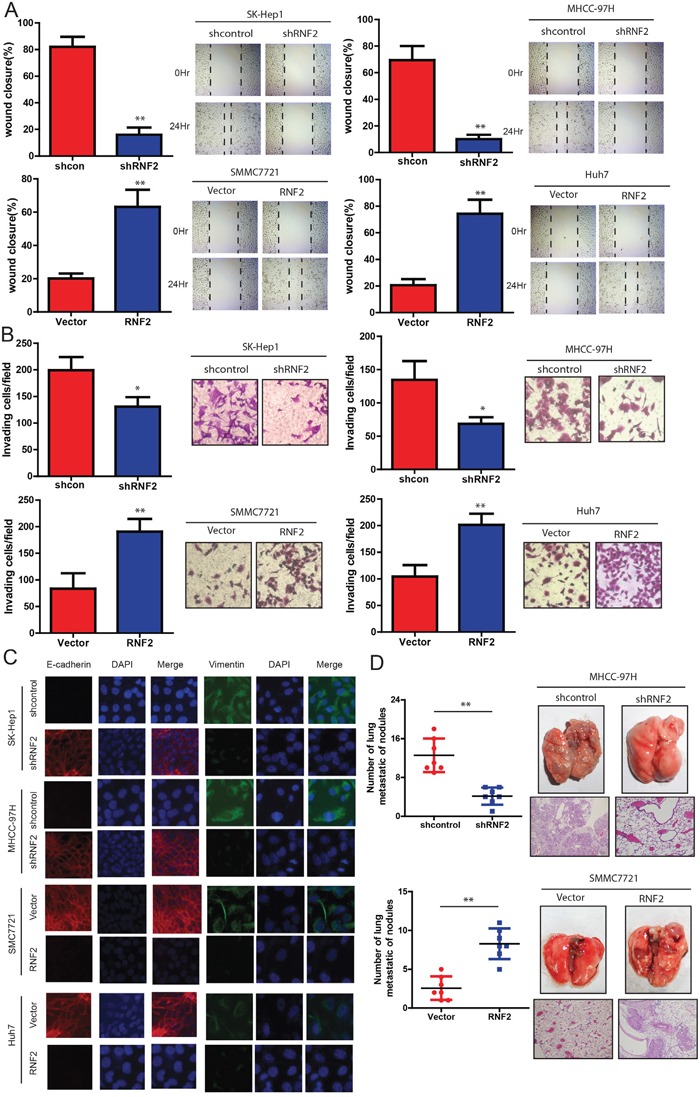
RNF2 knockdown suppresses invasion and metastasis of HCC cells HCC cell lines infected with RNF2 expression vector or its shRNA or control were used in the following studies. **A**. The wound-healing assay was used to explore the effect of RNF2 on the motility of HCC cells. The percentage of wound healing is shown in the diagrams (left panel). **B**. Effects of RNF2 knockdown or over-expression on HCC cell invasion in indicated cells *in vitro* using transwell assay. The diagrams were shown (left panel). **C**. Representative immunofluorescence (IF) images indicated that RNF2 has an effect on the expression of EMT genes in HCC cells. **D**. Effects of RNF2 over-expression on tumor metastasis of indicated cells in nude mice (*n* = 10 per group): the number of metastatic nodules in the lung (left-panel); representative morphological observation of lung metastases (right-upper panel); and histopathological observation of lung sections (right-lower panel). The results are presented as are means ± SD (*n* = 3 for each panel). Statistical significance was concluded at *P < 0.05, **P < 0.01, ***P < 0.001; # represents no statistical significance.

Next, we examined the effects of RNF2 on the ability of HCC cells to invade and migrate. It was found that the level of RNF2 expression was positively correlated with the rates of wound healing: higher levels of RNF2 expression with faster healing (Figure 4A). Similarly, SMCC7721 and Huh7 cells overexpressing RNF2 were found to have significantly higher rate of invasion than control cells, whereas SK-Hep1 and MHCC-97H cells expressing shRNF2 invaded slowly (Figure 4B). Consistently, based on immunofluorescence staining, we also observed a marked change in the expression of hallmark EMT genes in RNF2-expressing cells (Figure 4C). Cells with relatively high RNF2 expression displayed a mesenchymal-like phenotype because the expression of the mesenchymal marker (Vimentin) was increased. Conversely, knockdown of RNF2 strongly up-regulated the epithelial marker E-cadherin. We next examined the effect of the abnormal expression of RNF2 on tumor metastasis *in vivo*. As shown in Figure 4D, the mice injected with the RNF2-knocked down HCC cells exhibited less visible lung metastatic nodules than the control group, while the one injected with RNF2 overexpression HCC cells has more metastatic nodules. These data was further confirmed by histological staining and a statistical analysis, indicating that over-expression of RNF2 strongly promoted the ability of HCC cells to establish lung metastases. Collectively, our data indicate that RNF2 strongly promotes HCC cell invasion and metastasis potentialities.

### RNF2 directly ubiquitinates and degrades SIK1

**Figure 5:**
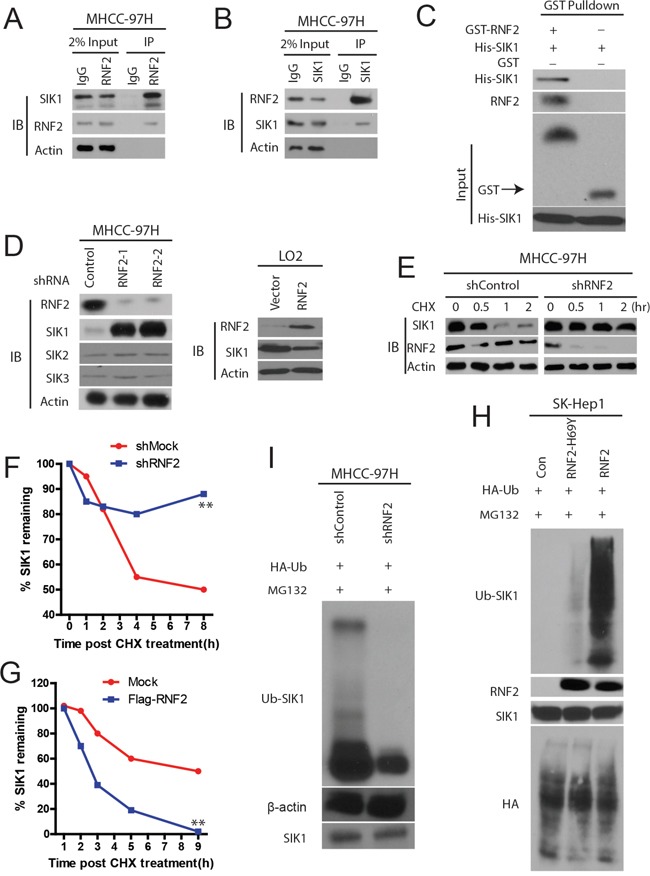
RNF2 is a E3 ligase of SIK1 and ubiquitinates SIK1 **A** and **B**. RNF2 interacts with SIK1 *in vivo*. HCC cell lysates were subject to immunoprecipitation with anti-IgG, anti-SIK1 (A), or anti-RNF2 (B) antibodies. The immunoprecipitates were subsequently blotted with the indicated antibodies. **C**. RNF2 interacts with SIK1 *in vitro*. SIK1 was purified from bacteria and incubated with GST or GST–RNF2 coupled to GSH–Sepharose. The proteins retained on the Sepharose beads were subsequently blotted with the indicated antibodies. **D**. The knockdown of RNF2 increases SIK1 protein levels, but not SIK2 or SIK3(left panel), RNF2 overexpression decreases SIK1 protein expression (right panel). **E**. HCC cells were transfected with the indicated shRNAs. After 72 h, the proteins were extracted and subjected to Western blot. **F** and **G**. RNF2 depletion or overexpression affects SIK1 stability respectively. HCC cells transfected with the indicated constructs were treated with CHX (0.1 mg/mL) and harvested at the indicated time. The cell lysate was subjected to Western blot analysis. **H** and **I**. ubiquitination assay of SIK1. Immunoblots of SIK1 and RNF2.

Next, we set out to characterize the molecular mechanisms of RNF2 in HCC progression. Given that there is obvious difference between the effects of RNF2-WT and RNF2-mutant, we reasoned that the impact of RNF2 on increasing HCC cell proliferation and invasion depends on its ubiquitination activity. Therefore, we hypothesized that RNF2 is an E3 ubiquitin ligase of SIK1. To test this idea, we purified recombinant RNF2 and SIK1 and performed coimmunoprecipitation assays of RNF2 and SIK1, which confirms the interaction of these proteins in HCC cells (Figure 5A and 5B). Pull-down assay in a cell-free system demonstrated that purified His-SIK1 bound to GST–RNF2, suggesting a direct interaction between RNF2 and SIK1 (Figure 5C).

Next, we investigated whether the binding of RNF2 regulates SIK1 activity. We found that RNF2 knockdown resulted in marked increases in the levels of endogenous SIK1 protein, but not SIK1 or SIK3 (Figure 5D), without affecting the expression of *SIK1* mRNA (data not shown). Interestingly, we found an decrease of SIK1 by RNF2 overexpression in normal liver cell line LO2 (Figure 5D). However, the proteasome inhibitor MG132 blocked the reduction of the SIK1 expression (data not shown), suggesting that RNF2 regulates SIK1 levels through proteasome-dependent degradation. Then we examined the degradation kinetics of SIK1 in the presence of the protein translation inhibitor cycloheximide (CHX) in HCC cells. It was found that in RNF2 knockdown HCC cells, the rate of SIK1 protein degradation was clearly reduced (Figure 5E and 5F), whereas RNF2 overexpression promoted SIK1 protein degradation (Figure 5G).

Then we examined whether RNF2 directly ubiquitinate SIK1. As shown in Figure 5H, the overexpression of RNF2 in HCC cells indeed markedly induced SIK1 poly-ubiquitination in the presence of the MG132, whereas H69Y-RNF2 mutant failed to increase the ubiquitination levels of SIK1 (Figure 5H). Conversely, the ubiquitination of SIK1 in RNF2 knockdown cells was significantly decreased (Figure 5I). Therefore, we believe that RNF2 is important for SIK1 ubiquitination and stability.

### RNF2 promotes HCC cell proliferation and invasion partially through SIK1 ubiquitination

**Figure 6:**
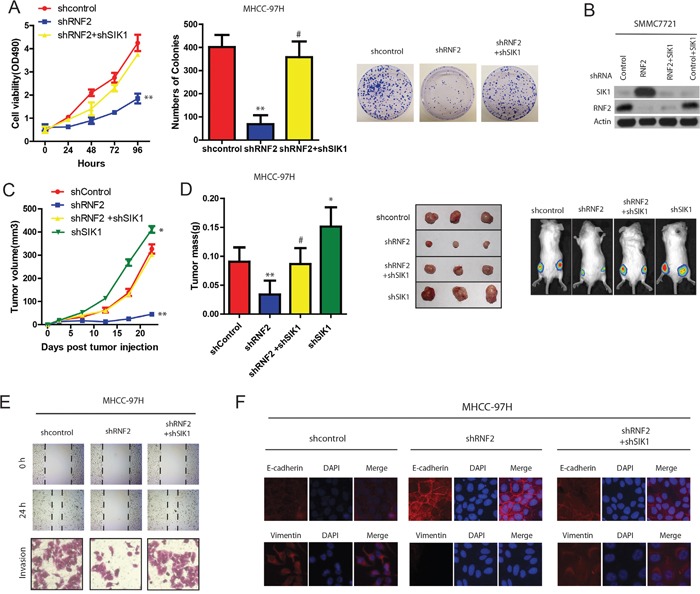
RNF2 promotes HCC cell proliferation and invasion partially through SIK1 ubiquitination **A**. Effects of RNF2 and SIK1 depletion on HCC Cell viability determined by MTT assays on HCC cells transfected with indicated plasmids or control(left panel). Effects of RNF2 and SIK1 depletion on HCC cell colony formation assays (right panel). **B**. Protein expression in HCC cells stably expressing the indicated shRNAs. **C**. Effects of RNF2 and SIK1 depletion on HCC tumor development. HCC cells were infected with indicated plasmids and injected subcutaneously into nude mice. Growth curve of tumor volumes. **D**. Estimated tumor weight for the indicated groups at day 20 after injection (left panel). (Right upper panel) representative photograph of xenograft tumors. (Right lower panel) noninvasive visualization of Luc-tagged tumor cells by whole-body fluorescence imaging showing a significant reduction in tumor size when RNF2 is depleted which was reverted by SIK1 knockdown. **E**. RNF2 promotes wound-healing and invasion through SIK1 signaling. Wound-healing and invasion assays for RNF2, shRNF2, and their control cells with/without SIK1 overexpression or shSIK1. **F**. RNF2 promotes EMT through SIK1 signaling. IF assay of E-cadherin and vimentin expressions in indicated treatment.

Next, we wanted to confirm hypotheses that the effects of RNF2 in HCC are exerted at least partially through SIK1 ubiquitination. As shown in Figure 6A and 6B, although the depletion of RNF2 in HCC cells suppressed HCC cell proliferation, SIK1 knockdown restored the cell proliferating ability resulting from the RNF2 depletion (Figure 6A and 6B). Importantly, The knockdown of RNF2 expression through RNA interference significantly reduced the growth of xenograft tumors, whereas knocking down both RNF2 and SIK1 expression reversed tumor growth to the level of the control group (Figure 6C and 6D). Conversely, HCC cells transfected with SIK1 expression vector partially reversed the oncogenic effect of RNF2 on tumor growth *in vivo* (data not shown).

To further study the role of RNF2 and SIK1 signaling in migration and EMT of HCC, we also examined the migrasive capacity of RNF2-interfered HCC cells with or without shSIK1 treatment. Wound-healing and transwell assays demonstrated that SIK1 knockdown obviously reverted the decreased-migration capacity of RNF2 knockdown cells (Figure 6E). IF analysis revealed that RNF2 knockdown increased E-cadherin expression, while both SIK1 and RNF2 knockdown increased vimentin expression in HCC cells (Figure 6F). These results strongly indicate that RNF2 is a SIK1-dependent key player in HCC progression, and RNF2 suppressed HCC growth and metastasis at least partiallybytargeting SIK1.

## DISCUSSION

Our previous study showed that SIK1 down-regulation accelerates the growth and invasion of HCC, and regulation of SIK1 occurs at a transcriptional and posttranscriptional level through protein stability [10, 17]. However, the upstream pathways regulating SIK1 stability in HCC are still not well understood.

In this study, for the first time, we investigated the impact of RNF2 on SIK1 expression and its role in HCC tumorigenesis. Our data indicate that: 1) A high DNA copy number of RNF2 gene significantly correlated with poor prognosis in HCC. 2) RNF2 overexpression is concurrent with SIK1 down-regulation in HCC patients. 3) Ectopically expressed RNF2 results in a decrease in SIK1 levels in HCC cell lines by accelerating its degradation; 4) RNF2 physically interacts with SIK1 and is an E3 ligase of SIK1, suggesting that RNF2 is an important upstream negative regulator of SIk1 in HCC. 5) RNF2-mediated SIK1 degradation may play an important role in liver cancer progression and resistance to chemotherapy. We believe that our identification of RNF2 as a specifically bound, negative regulator of SIK1 contributes to our understanding of how transcriptional regulation and ubiquitination comprise a unified molecular mechanism to precisely tune SIK1 activity in HCC cells.

The ubiquitin-proteasome system has become a critical target of deregulation leading to HCC [18]. It has been reported that the stability of SIK1 in primary myogenic progenitor cells is tightly and differentially regulated by ubiquitylation and proteasome-mediated degradation [17]. Another study also identified a functional PEST domain near Thr475 that contributes to SIK1 degradation [19]. Although SIK1 was found to co-immunoprecipitation with smurf2, this E3 ligase has not been shown to be responsible for regulating the cellular level of SIK1 by ubiquitylation and proteolysis [20]. In this study, for the first time, we showed that down-regulation of SIK1 was inhibited in HCC cell lines with RNF2 knockdown, and that exogenous RNF2 decreased the stability of SIK1, which indicates that RNF2-mediated SIK1 degradation is dependent on the proteasome pathway. Thus, these findings identify a novel pathway regulating SIK1 in HCC independent of the transcriptional pathway.

Several clinical studies have shown that RNF2 is upregulated in several types of human cancer, including breast, nasopharyngeal, pancreatic, and lung carcinomas, which are associated with increased tumor growth and poor outcome [21, 22]. These data suggest that RNF2 is a tumor promoter. Hsu et al. reported that RNF2 was overexpressed in HCC [23]. However, very little was known about the action mechanisms of RNF2 in HCC. Previous studies have shown that RNF2 negatively correlated with p53 level in HCC tissues [24]. However, we demonstrated that there was no significant increase in p53 levels by RNF2 depletion in some HCC cell lines. Our data indicate that silencing p53 and RNF2 does not abolish the inhibitory effect of tumor growth-induced RNF2 depletion. In addition, we did not observe an increase in p27 after RNF2 silencing. On the contrary, our findings confirmed that restoration of SIK1 induced by RNF2 depletion could inhibit cell growth and induce cell apoptosis in HCC cells, and that SIK1 siRNA can counteract the inhibitory effect of RNF2 lost on tumor growth. Ongoing preclinical studies from our research will explore the efficacy of these novel treatment combinations in well-established animal models of HCC carcinogenesis.

In summary, our findings from the present study clearly indicate that RNF2 is a novel regulator of SIK1 and promotes its degradation in HCC pathogenesis. Moreover, our study uncovers that overexpression of RNF2 not only plays an important role in the pathogenesis of HCC, but it might also serve as a useful prognostic molecular marker indicative of poor outcome in the individual patient.

## MATERIALS AND METHODS

### Cell culture and antibodies and reagents and transfection

Human HCC cells (Hep3B, and HepG2, SMMC7721, MHCC97H, HCCLM3 and Huh-7) and HEK293T cell lines were purchased from the American Type Culture. And these cell lines were cultured in DMEM supplemented with 10% FBS and 1% penicillin-streptomycin. All cell cultures were maintained at 37°C in a humidified incubator with 5% CO_2_. Anti-RNF2 (ab3832 and ab28629) antibodies were purchased from Abcam. Anti-FLAG, anti-HA, anti-Lamin, anti-Tubulin, and anti-Actin antibodies were from Sigma. CHX (C4859), and puromycin (P8833) were from Sigma. MG132 was from Calbiochem. Transfections were performed in 35×10–mm tissue culture dishes by using 1.0 μg of DNA and 2.5 μl of Lipofectamine 2000 (Invitrogen). Immunofluorescence or immunoprecipitation experiments were performed 24 hours later.

### HCC patients sample

All tissues enrolled in this study were collected from patients undergoing HCC resection from January 2007 to May 2013 at the First Hospital of Jilin University. The diagnosis of HCC was according to World Health Organization criteria. The clinical typing of tumors was determined based on the International Union against Cancer tumor-node-metastasis classification system. The liver function was evaluated by Child-Pugh score system. Tumor differentiation was determined according to the Edmondson grading system. HCC patients are divided into two groups: high RNF2 and low RNF2. The level of RNF2 expression to perform this classification is considered only in the tumor part and not in the paired non-tumour tissue. In addition, the expression of RNF2 is quantified at the protein level using immunohistochemistry. The average age of these patients was around 49 years old. All of them are HBV negative without liver cirrhosis and with the level of AFP in the normal range. Ethical approval for the use of human subjects was obtained from the Research Ethics Committee of the First Hospital of Jilin University with the informed consent of patients.

### MTT assay

About 500 or 1000 HCC cells in 100 ul medium were added into the 96-well microplate. After the cells had been either treated as indicated, 10 ul MTT solution (5 mg/ml, Sigma-Aldrich) was added to each well for 3 h. The medium was carefully removed by pipetting without disturbing the formazan crystals at the bottom. To dissolve the crystals, 100 ul DMSO was added, which was followed by absorbance measurement at 570 nm.

### Wound healing assay

In brief, cells were plated in 6-well plates. After growth to about 70–80% confluency, cells were transfected with indicated plasmids according to manufacturer's protocol, and cultured until almost totally confluent. Cell motility was then determined by the assay in which a scratch was made along the axis of the plate using a pipette tip. Cells were washed twice with PBS to remove floating cells and complete medium with 5% FBS was added after that. Migration of cells into the scratch was photographed and the wound distances were measured at 0 and 24 h under light microscope at 10× magnification.

### Colony forming assay

Briefly, 2000 indicated HCC cells were seeded into 6-well microplate and fresh medium was replenished every 3 days. Then the medium was removed and washed with PBS. Then HCC cells were stained 6% glutaraldehyde (Sigma-Aldrich) and 0.5% crystal violet solution (Sigma-Aldrich) for 20 mins. Finally, the plate was carefully rinsed with water to remove excessive stain. The plate was left to air-dry at room temperature followed by colony count.

### Immunohistochemical staining

Immunohistochemical (IHC) staining was performed as previously described using anti-SIK1 and anti-RNF2 ((Santa Cruz Biotechnology, USA). After washing, sections were incubated with a fluorophore-linked secondary antibody (Alexa Fluor 488–anti-rabbit IgG and Alexa Fluor 568–anti-mouse IgG; A10468, A10494; Life Technologies). After staining, slides were mounted in Aqua Poly/Mount (Polysciences Inc.) and photographed under an AxioVision IIe microscope with a digital camera.

### Flow cytometric analysis

Indicated cells were collected, fixed in 80% ethanol and stored at 4°C. Then the cells were washed and suspended in 10 ml of PBS and 1ml of RnaseA solution (250 mg/ml) followed by incubation for 30 minutes at 37°C. Then, 100 μl of propidium iodide stain (100 mg/ml) was added to each tube and incubated at 4°C for 30 minutes prior to analysis. Flow cytometric analysis was conducted using BD LSRFortessa (BD Biosciences). The percentages of the cells in different phases of the cell cycle were analyzed by using FlowJo software.

### Ubiquitination assay

Indicated cells were transfected with indicated plasmids. Cells were treated with MG132 for 6 h before harvesting, and lysed with RIPA buffer supplemented with 10 mM iodoacetamide (GE Healthcare) and protease inhibitors. Endogenous SIK1 was immunoprecipitated with 1 μg of anti-SIK1 polyclonal antibody and immunoblotted with anti-HA or indicated antibodies. Or a 100 μL sample of the *in vitro* translated FLAG-tagged RNF2 was purified using Flag beads, and the reconstituted complex was used for *in vitro* ubiquitination without elution from the Flag beads. 2 μL *in vitro* translated 35S-labeled SIK1 was incubated in a 30 μL reaction mixture containing 5 mM MgCl2, 0.6 mM DTT, 2 mM ATP, 50 mM Tris·HCl (pH 7.5), 100 ng of ubiquitin-activating enzyme E1, 200 ng of ubiquitin-conjugating enzyme UbcH5c, 10 μg of ubiquitin (Calbiochem), and ∼2 μg of RNF2. After incubation at 37 °C for 2 h, the reactions were boiled and separated on SDS/PAGE gel.

### Quantitative real-time PCR (qRT-PCR)

Total RNA was extracted from HCC cell lines or fresh frozen tumor specimens by using Trizol reagent (Invitrogen, CA) according to the manufacturer's instructions. qRT-PCR was performed using the SYBR^®^ Green Realtime PCR Master Mix assay kit (Toyobo, Japan) according to the manufacturer's instructions. The results were analyzed using the 2^−ΔΔCt^ method as the following formula: ΔΔCt = ΔCt_HCC_ −ΔCt _ANLT_, ΔCt = Ct_JARID2_ −Ct_GAPDH_. Primers for qRT-PCR. RNF2: forward-GTGTGTGGATGTGTGTTT, reverse-ATGTGGCTATGTATATGTTACC; GAPDH: forward-TCCTGGTATGACAACGAAT, reverse-TCTTCCTCTTGTGCTCTT; and Puma: forward-TGTGAATCCTGTGCTCTG, reverse-CCCAAATGAATGCCAGTG. RNA Interference. RNF2 control shRNA, RNF2 shRNAs, and SIK1 shRNA fragments were cloned into the pSUPERretro vector. The following shRNA sequences were used: Control shRNA sequence, TCGGTACTCAACCGTTAAG; RNF2 shRNA-1, ACGGAACTCAACCATTAAG; RNF2 shRNA-2, TGGATGGTGCTAGTGAAAT. The shRNA plasmids were transfected into cells as described. The lentiviral shRNA expression system to knock-down human SIK1 is commercially available from Sigma (SHDNA MISSION® shRNA Plasmid DNA; St. Louis, USA). RNF2 cDNA was cloned into pRK5-FLAG vector or pGEX-4T1 vector. The RNF2 point mutants were generated using a site-directed mutagenesis kit (Transgen). The complementary DNA (cDNA) clone of SIK1(Origene) was subcloned into pWzl-Blast by PCR with primers containing His epitope–encoding sequence to generate His-tagged SIK1.

### Immunofluorescence

Indicated HCC cells (5 × 104 cells) were fixed with 4% paraformaldehyde, permeabilized with 0.1% Triton X-100 in PBS, and blocked with 3% BSA in PBS. Expression of E-cadherin, vimentin, ZO-1, N-cadherin, Twist1, SIK1, and β-catenin was detected using each respective primary antibody and visualized with Alexa Fluor 488-conjugated secondary antibodies (Invitrogen, USA). Nuclei were counterstained with Hoechst 33258. All scale bars represent 200 μm. All images were captured by DeltaVision RT wide-field epifluorescence microscope imaging system and analyzed by softWoRxs image analysis program (Applied Precision, USA).

### GST pulldown

GST or His fusion proteins were purified following the manufacture standard protocol. GST–RNF2 fusion proteins bound to the GST Sepharose beads and then incubated with purified His-SIK1. After washing for five times, the bound proteins were separated by SDS-PAGE and probed with indicated antibodies.

### Western blotting

Tissues or cells were lysed in lysis buffer. A BCA assay kit (Beyotime, China) was used to measure protein concentration of the lysates. Protein samples were electrophoresed on SDS–polyacrylamide gels and transferred to nitrocellulose membranes. The membranes were blocked with 5% skim milk in Tris-buffered saline containing 0.1% Tween-20 (TBST) and subsequently incubated with primary antibody overnight at 4°C. At room temperature, the membranes were washed again with TBST for three times, and then incubated with a secondary antibody for 1h, followed by three times TBST washing for 5 min. Protein bands were visualized with the Super Signal Reagents (Pierce, USA).

### Co-immunoprecipitation

Cells were lysed in ice-cold RIPA buffer supplemented with protease inhibitors. After the insoluble fraction was removed by centrifugation at 4°C for 10min at 12,000rpm, the lysates were pre-cleared using protein-A/G sepharose. An equal amount of normal IgG was used as negative control. Incubate the above lysates with protein-G sepharose after adding 2μg of the indicated primary antibodies at 4°C overnight, immunoprecipitation was performed. After extensive washing, the sepharose beads were boiled in 50μl 1 × SDS loading buffer for 5min at 95°C. The eluted proteins were then subjected to western blotting.

### Cell proliferation

According to the manufacturer's protocol, cell proliferation was determined by using cell counting assay Kit-8 (CCK-8) (Dojindo, Japan). In brief, 5 × 10^3^ cells/well were cultured in 96-well flat-bottomed microplate. Cells were incubated in 10 % CCK-8 (Dojindo) diluted in normal cultured medium at the indicated time, and then were incubated for 1 h at 37 °C. The absorbance of each well was measured with a microplate reader set at 450 nm. The experiment was repeated three times.

### Cell migration and invasion assay

Cell migration and invasion were determined by wound healing assay and invasion chamber assay, respectively. For wound healing assay, transfected cells (10^4^/well) were cultured in six-well plates for 24 h, and then a linear scratch wounds was created on the confluent cell monolayers using a sterile plastic micropipette tip. Cells were cultured in RPM1640 or DMEM medium including 10 % FBS. At 0 and 24 h after the wounding, cells were imaged. Individual cells were quantified as an average of at least five randomly selected fields for each experiment. For invasion assay, 2 × 10^4^ transfected cells were placed into the upper side of the polycarbonate transwell filter coated with Matrigel (BD Biosciences), and cultured in serum-free RPM1640 or DMEM medium. Medium containing 20 % FBS was added to the lower chamber. Those left on the upper chamber were carefully wiped out with cotton swab after 48 h,, and cells located on the lower surface of the chamber were fixed with 70 % ethanol for 30 min and stained with 0.2 % crystal violet for 10 min. Photographs of five randomly selected fields of the fixed cells were taken and counted using a microscope (Olympus, Japan). These experiments were repeated three times.

### Cell cycle analysis

Indicated HCC cells were harvested 72 h after indicated treatment. Then the cells were fixed in 75% ethanol overnight at –20°C after washed with PBS. Then the cells were washed with PBS for twice and applied with DNA Prep Stain (Beckman Coulter, USA) and RNase for 30 min. Next, the cell cycle analysis was performed by Flow Cytometry System with FACSDiva software. The data were analyzed by ModFit LT 3.2 software (Verity Software House, USA) and the cell cycle distribution was described as the percentage of cells in G1, S, and G2 populations.

### Xenograft mice model

Animal experiments were performed according to the National Institute of Health Guide for the Care and Use of Laboratory Animals and approved by the Scientific Investigation Board of the Hospital. Balb/c athymic nude mice were obtained from the Vital River Animal Laboratories (Beijing, China). Indicated cells were suspended in 0.1ml PBS, then injected subcutaneously into the side of the posterior flank of the same female BALB/c athymic nude mice at 4 weeks old. Tumor growth was measured daily using calipers. According to the formula volume 1/4 length×width^2^×0.5, the tumor volume was calculated. Additionally, 1 × 10^6^ cells per mouse were injected into the tail veins of 6- to 8-week-old male athymic nu/nu mice. Then metastasized loci and tumor growth were monitored using the IVIS 100 Imaging System. The mice were humanely euthanized 8 to 10 weeks after implantation, and their lung tissues were collected for standard histological examination. Tumor formation and metastasis were imaged by bioluminescence. And bioluminescence was detected with Berthold NIGHTOWL LB983 imaging machine. Each mouse was photographed, and then luminescent images were acquired with various (1-60 seconds) exposure times. The resulting grayscale photographic and pseudocolored luminescent images were automatically superimposed by the Living Image software to match the observed luciferase signal with its location on the mouse.

### Statistical analysis

All data were analyzed using the statistical software SPSS 18.0 for Windows (SPSS Inc., IL). The DFS and OS rates were calculated using the Kaplan-Meier method, with the log-rank test applied for comparison. Spearman's rank analysis was used to analyze the correlations between different protein expressions level. The Student's *t*-test, χ^2^ test or Fisher's exact test was used for comparisons between groups. Two-sided *p*-values were calculated, and statistically significant data are indicated by asterisks *P* < 0.05 (*), *P* < 0.01(**), *P* < 0.01(***). The results are expressed as mean ± standard deviation (SD) from at least three independent experiments.

## SUPPLEMENTARY MATERIALS AND TABLES



## Abbrevations

SIK1: salt-inducible kinase 1
HCC: hepatocellular carcinoma
LKB1: liver Kinase B1
qRT-PCR: quantitative real-time PCR
