# Supplementary material for: Down-regulation of salt-inducible kinase 1 (SIK1) is mediated by RNF2 in hepatocarcinogenesis
Source: Oncotarget. 2016 Nov 29;8(2):3144–55. doi: 10.18632/oncotarget.13673 (PMC5356871; doi:10.18632/oncotarget.13673)
Supplement: Supplementary file 1 [file oncotarget-08-3144-s001.pdf]

Down-regulation of salt-inducible kinase 1 (SIK1) is mediated by RNF2 in hepatocarcinogenesis

SUPPLEMENTARY TABLE

Supplementary Table S1: Correlation Between the Prognostic Factors and Clinicopathologic Characteristics in HCC (Cohort 1, n = 67)

	RNF2 expression in HCC			SIK1 expression in HCC		
	Low	High	p Value	Low	High	p Value
**All cases**	43	24		27	40	
**Gender**						
Women	21	11	NS	15	25	NS
Men	22	13		12	15	
**Age**						
<50	20	12	NS	13	22	NS
>50	23	12		14	18	
**HBV infection**						
Negative	25	10	NS	16	25	NS
Positive	18	14		11	15	
**AFP**						
Negative (<20 ng/ml)	28	12	NS	14	20	NS
Positive (>20 ng/ml)	15	12		13	20	
**Cirrhosis**						
Negative	22	13	NS	16	29	NS
Positive	21	11		11	11	
**HBV history**						
No	24	15	NS	17	30	NS
Yes	19	9		10	10	
**Family history of HCC**						
No	21	12	NS	15	24	NS
Yes	22	12		12	16	
**Capsular Invasion**						
No	32	18	**<0.01**	19	28	**<0.01**
Yes	11	6		8	12	
**Tumor size**						
<	38	9	**<0.01**	8	28	**<0.01**
>	5	15		19	12	
**Pathological differentiation**						
I–II	22	13	NS	13	29	NS
III–IV	21	11		14	11	
**TNM clinical stage**						
I-II	19	11	NS	17	25	NS
III	24	13		10	25	

Abbreviations: Correlation between SIK1, RNF2 expression and clinicopathological characteristics of 67 patients with HCC Chi-square test was used.
Bold text represents p values with significant difference.
HBV, hepatitis type B virus; AFP, α-Fetoprotein; HCC, hepatocellular carcinoma.
